# Case Report: Cutaneous Pleomorphic Lymphangiosarcoma in a Dog Exhibiting Features of Human Composite Hemangioendothelioma

**DOI:** 10.3389/fvets.2021.666226

**Published:** 2021-10-22

**Authors:** Matthew R. Cook, Joshua N. Lorbach, Mary E. White, Geoffrey J. Zann, Rachel E. Cianciolo, Laura E. Selmic, Vincent Wavreille, William C. Kisseberth

**Affiliations:** ^1^Department of Veterinary Clinical Sciences, College of Veterinary Medicine, The Ohio State University, Columbus, OH, United States; ^2^Department of Veterinary Biosciences, College of Veterinary Medicine, The Ohio State University, Columbus, OH, United States; ^3^Department of Population Medicine, College of Veterinary Medicine, Midwestern University, Glendale, CA, United States

**Keywords:** lymphangiosarcoma, composite hemangioendothelioma, cytology, histopathology, canine

## Abstract

**Background:** Angiosarcomas are a broad category of vascular origin neoplasms that are poorly characterized in veterinary species. Lymphangiosarcoma (LAS) is an uncommon type of angiosarcoma reported in humans and canines arising from lymphatic endothelium. LAS can be differentiated from other angiosarcomas in dogs based on expression of Prospero-related homeobox gene-1 (PROX-1) or lymphatic vessel endothelial receptor-1 (LYVE-1). Composite hemangioendothelioma (CHE) is a rare angiosarcoma subtype described in people and characterized by a variable biologic behavior and infrequent metastasis. This variant of angiosarcoma histologically combines features of retiform hemangioendothelioma and epithelioid hemangioendothelioma. Information regarding the cytologic and histopathologic appearance and clinical course of dogs with vascular tumors that exhibit features of CHE are unknown. Here, we report a case of pleomorphic LAS with features of CHE arising in a dog and treated with surgery and adjuvant chemotherapy.

**Case presentation:** A 10-year-old intact male Labrador retriever presented with an approximately 6-cm-diameter cutaneous mass caudal to the left elbow that was progressively growing over 1.5 years. On physical examination, palpable extensions were identified coursing proximally over the triceps with concurrent loco-regional peripheral lymphadenopathy. Fine needle aspirates (FNA) and cytologic assessment of the cutaneous mass, left prescapular, and accessory axillary lymph nodes reported that this appeared to be a metastatic epithelial neoplasm, although a mixed carcinoma or collision tumor could not be excluded. An incisional biopsy of the mass was submitted for histopathology and was consistent with a well-differentiated angiosarcoma with features of CHE. The neoplasm expressed vimentin, CD31, von Willebrand factor (vWf), and PROX-1, supporting the diagnosis of LAS. Complete staging was performed, and no additional metastatic lesions were identified. Left forelimb amputation and lymph node removal were performed. Based on the diagnosis of metastatic LAS, doxorubicin chemotherapy was administered. 7 months post-amputation, the tumor recurred at the amputation site without evidence of metastatic disease.

**Conclusion:** This report describes a malignant, locally aggressive lymphatic origin vascular tumor in a dog, with features consistent with descriptions of CHE in humans. Cytologic features in this case were discordant with its true mesenchymal etiology, obfuscating diagnosis. The morphologic features of the mesenchymal neoplastic population and immunohistochemistry (IHC) labeling ultimately supported a diagnosis of pleomorphic LAS with features of CHE.

## Background

Angiosarcomas comprise a diverse group of tumors arising from either vascular or lymphatic endothelium. Lymphangiosarcoma (LAS) is a rare, locally aggressive tumor in the dog originating from the endothelium of lymphatic vessels (1). While LAS is commonly associated with a history of chronic lymphedema secondary to lymph node extirpation, mastectomy, irradiation, or trauma in humans, these risk factors are not commonly reported in the veterinary literature (2, 3). Diagnosis of LAS in the dog is typically determined by histopathologic evaluation in conjunction with IHC expression of PROX-1 or LYVE-1, both markers of lymphatic endothelium, to help further classify less well differentiated vascular tumor (1). LAS is associated with a poor prognosis in humans with a reported survival between 5 and 8 months (3). While rarely reported in the dog, a case series of 12 dogs reported variable survival times ranging from 60 to 941 days with variable treatments, although affected dogs treated with multimodal therapy tended to have better outcomes in one study (4).

Hemangioendothelioma (HE) is a well-described subset of vascular tumors in humans. These malignancies typically demonstrate a wide range of biologic behavior (5). HEs are typified by their ability to recur locally; however, their metastatic potential varies based on the pathologic subtype. In particular, composite hemangioendothelioma (CHE) is a rare subtype of low-to-intermediate grade angiosarcoma that microscopically is composed of a mixture of benign, intermediate, and malignant vascular components (6–9). CHEs demonstrate some histologic variability because their morphology consisting of a combination of at least two HE subtypes, with retiform hemangioendothelioma (RH) and epithelioid hemangioendothelioma (EH) being the most common combination of subtypes. RHs are histopathologically unique in that they are characterized by elongated, anastomosing vascular channels resembling the rete testis, with the vascular channels lined by a single layer of cuboidal endothelium with a distinctive “hobnail” appearance (10). RH also lack atypical mitoses and significant necrosis, helping to distinguish this neoplasm from other variants of HE. Additionally, RH usually expresses CD31, CD34, vWf, and commonly associated lymphocytic infiltrates express the lymphocyte markers CD3+ and CD20+ (11). While typically locally aggressive, metastatic disease is rare, occurring in <10% of cases in humans (12). Cases with regional lymph node metastasis have been reported, and while rare, this appears to be the most common metastatic pattern reported for RH (10, 12). In contrast to RH, EHs are a heterogeneous subset of HE exhibiting variable biologic behavior, ranging from indolent to highly metastatic behavior involving the lungs or bone (13). Histopathologically, EH are typically arranged in cords and nests associated with hyaline or myxoid stroma, and can present with spindle-shaped tumor cells (13).

While this spectrum of vascular neoplasms has been relatively well described in people, HE is rare in dogs. Hemangiosarcoma, however, is a very commonly diagnosed entity in the dog, primarily arising in the spleen, right atrium and auricle, skin and subcutaneous tissues (14). To date, only two cases of canine angiosarcomas with features of human RH have been described (15). Histopathologic features of these cases included the presence of elongated vascular channels, bulging hobnail nuclei, and marked lymphoplasmacytic inflammation. The neoplasms from both dogs described in these reports expressed vWf and CD31. In comparison with the human correlate, these two cases were more aggressive, as disseminated metastatic disease was present. In one case that was diagnosed at necropsy, metastatic lesions were present within the right atrium, spleen, lungs, brain, and cervical spinal cord (15). Here, we describe the first reported case of a pleomorphic LAS with features of CHE described in the dog.

## Case Description

**Figure 1:**
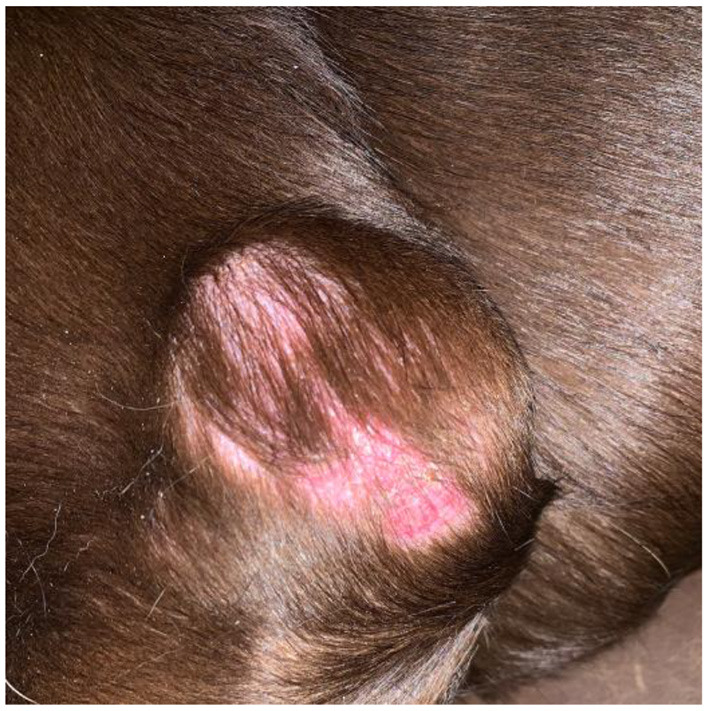
Image of the primary mass located on the left lateral elbow obtained approximately 1 month prior to presentation. Note the overlying alopecia and discoloration of the skin.

A 10-year-old, 36.4-kg male intact Labrador retriever presented to The Ohio State University Veterinary Medical Center for evaluation of a slowly growing cutaneous mass. The owners reported a flat, cutaneous mass caudal to the left elbow that slowly increased in thickness and alopecia over a period of 1.5 years prior to evaluation. Prior to presentation, the mass was aspirated by another veterinarian and was diagnosed as a carcinoma based on cytology. Physical examination identified a semi-fixed, 6 cm, raised cutaneous mass (Figure 1). Additionally, the left prescapular and axillary lymph nodes were firm and enlarged on palpation, measuring 4.4 and 2.9 cm in diameter, respectively (Figure 1). A complete blood count and serum chemistry panel revealed no values outside the laboratory reference intervals. Thoracic radiographs revealed no evidence of pulmonary metastatic disease or other clinically relevant abnormalities.

**Figure 2:**
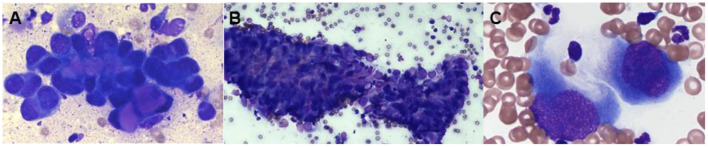
Representative photomicrographs of a fine-needle aspirates from the left elbow mass **(A)** and the left prescapular lymph node **(B,C)**, modified Wright–Giemsa stain. **(A)** The cytology image shows a population of neoplastic cells found in tight aggregates and occasionally individually. The neoplastic cells often contained eosinophilic material within the cytoplasm, ×100. **(B)** There is a population of neoplastic cells that are cytologically similar to the population seen in the left elbow mass, × 20. **(C)** The neoplastic cells occasionally were seen individually and exhibited a spindloid appearance, × 100.

FNA samples from the elbow mass and left superficial cervical lymph node were collected and submitted for cytologic evaluation. Cytology from the primary elbow lesion was suggestive of a carcinoma with moderate, mixed inflammation, evidence of previous hemorrhage, and reactive fibroplasia, although initial report noted that carcinosarcoma or mixed-type carcinoma could not be excluded. Multiple clusters of epithelial cells frequently containing intracellular, eosinophilic secretory material were found in acinar formations, which was suggestive of a glandular neoplasm (Figure 2). A similar neoplastic cell population was seen in the lymph node sample.

One week following initial presentation, the dog was presented to The Ohio State University Veterinary Medical Center for further staging. The owners reported that the dog did not exhibit any signs of discomfort, lameness, or signs of systemic disease. On physical examination, a firm, flat, mobile, subcutaneous, alopecic, 4.7 × 5.0 cm subcutaneous mass was palpable caudal to the left elbow, with firm subcutaneous extensions coursing proximally over the triceps. The left superficial cervical and axillary lymph nodes were also firm and enlarged on palpation. An abdominal ultrasound did not identify any clinically relevant abnormalities. An ultrasound guided FNA of the left accessory axillary lymph node was performed and submitted for cytologic evaluation. The sample was moderately cellular with moderate numbers of neoplastic cells found in clusters often associated with eosinophilic matrix material and occasionally found individually. Occasional spindle-shaped neoplastic cells were observed (Figure 2). Round to irregular nuclei had fine to coarse chromatin with one to four round to irregular, distinct nuclei. The cytologic impression was that this neoplasm was malignant. Neoplastic cells seen on the FNA of the left axillary lymph node appeared cytologically similar to the primary tumor, supporting regional metastasis.

An incisional wedge biopsy was then performed and submitted for histopathologic evaluation. The initial histologic diagnosis based on H&E staining was a well-differentiated angiosarcoma. Immunohistochemistry was subsequently performed, revealing strong labeling for vimentin (mouse monoclonal, 1:100, Dako cat. M0725) and CD31 (rabbit polyclonal, 1:50, Abcam cat. 28,364). These findings in conjunction with absent labeling for cytokeratin (mouse monoclonal, 1:50, Abcam cat. 86,734) were supportive of the diagnosis of angiosarcoma. Epithelioid hemangiosarcoma was also discussed as a differential diagnosis based on cellular morphology in some regions of the neoplasm.

**Figure 3:**
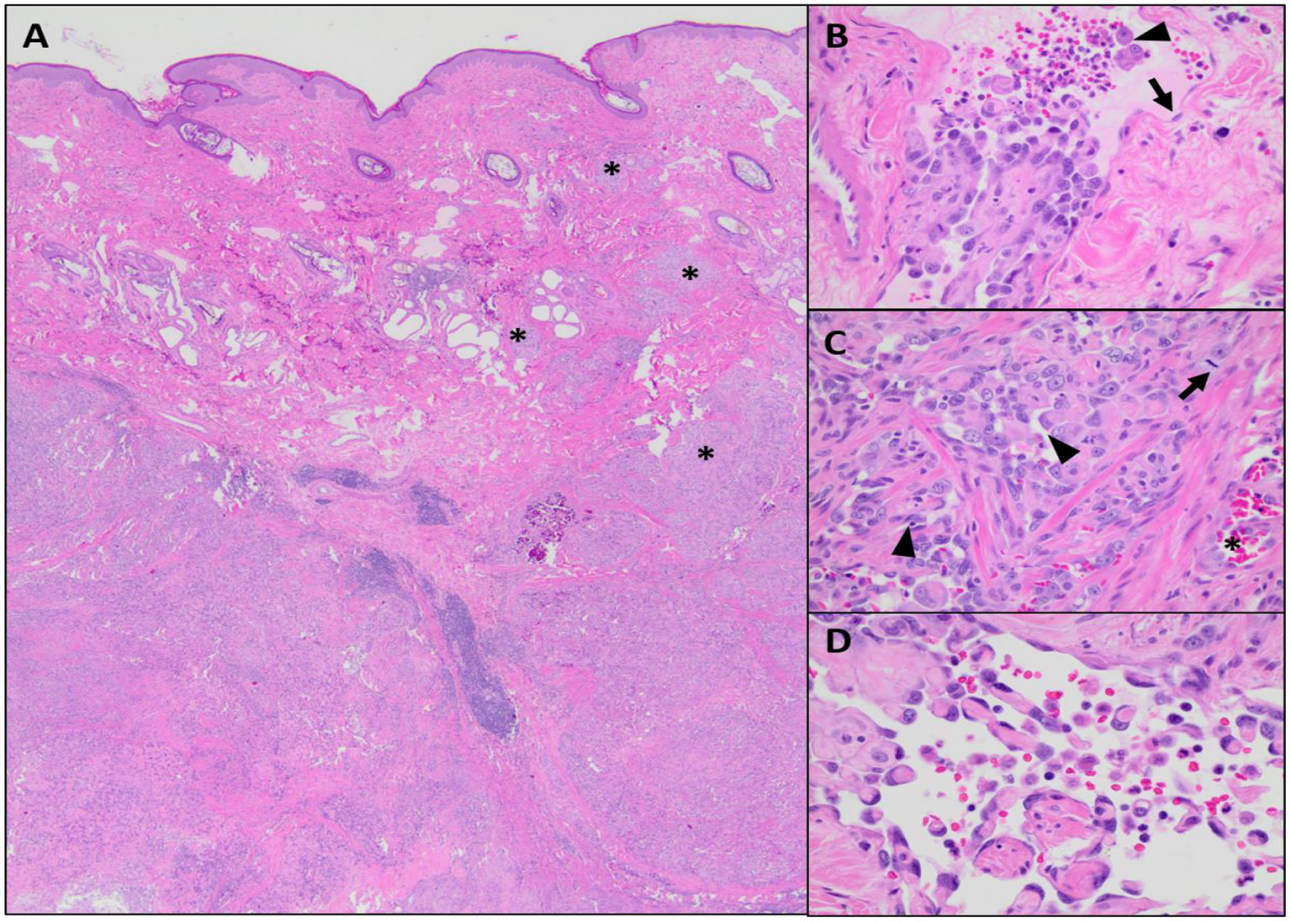
Microscopic features of the neoplastic population, hematoxylin and eosin (H&E). **(A)** The neoplastic population is centered within the deep dermis but invades overlying mid to superficial dermis in multiple areas (asterisks), × 20 magnification. **(B)** There are solid aggregates of neoplastic cells along with occasional individualized cells (arrowhead) located within endothelium-lined (arrow) vascular profiles, × 40 magnification. **(C)** The majority of the neoplasm consist of disorganized sheets of pleomorphic, plump, epithelioid cells with moderate mitotic activity (arrow); intercellular channels are mostly compressed (arrowhead), but occasionally contain erythrocytes (asterisk), 40 × magnification. **(D)** Scattered throughout the neoplasm, there are regions with delicate, anastomosing, hyaline eosinophilic trabeculae lined by relatively flattened endothelial cells forming rete-like vascular profiles, × 60 magnification.

Following microscopic diagnosis, a computed tomography (CT) scan with contrast [Iohexol, 2 ml/kg with max dose of 30 ml, intravenously (IV), Omnipaque™, GE Healthcare] was performed. Other than the mass, its subcutaneous extensions, and the two previously noted metastatic regional lymph nodes, no pulmonary or other lesions concerning metastatic disease were identified. A left forequarter amputation was performed, and the left prescapular and left axillary lymph nodes were extirpated. The forelimb and both lymph nodes were submitted for histopathology. Histologic findings in the primary mass sampled following amputation were similar to that of the previous incisional biopsies of the tumor. Compared with the incisional biopsy, the limb amputation permitted more extensive sampling of the mass, revealing heterogeneity within the neoplasm in terms of both cell morphology and vascular channel formation. In the amputation specimen, the deep dermis contained an unencapsulated, locally invasive, malignant mesenchymal population forming expansile nodules with random intervening bands of collagen, multifocal central tumor necrosis, and reactive mixed inflammation (Figure 3A). Neoplastic cells were supported by a fine fibrovascular stroma and arranged in narrow bands, sheets, and disorganized vascular profiles. Individual cells were plump and polygonal to spindle-shaped with indistinct cytoplasmic borders. Cells were supported by, and often encircled, narrow to thick eosinophilic trabeculae (wrap-around cells), forming a meshwork of vascular channels containing variable numbers of erythrocytes, leukocytes, and individualized neoplastic cells (Figure 3B). Many regions of the neoplasm were devoid of erythrocytes, appearing instead as disorganized sheets of neoplastic cells intermixed with empty, narrow clefts and channels (Figure 3C). These more densely cellular regions contained occasional aggregates and pearls of myxohyaline eosinophilic stroma intermixed with the neoplastic population. Occasionally, the vascular profiles within the neoplasm exhibited distinct retiform architecture (Figure 3D). Nuclei were plump and oval to indented, with pale basophilic chromatin and multiple prominent nucleoli. Nuclear atypia and anisocytosis were moderate to marked. Rare binucleation was observed, and there were five mitoses in 10 × 40 fields. Bordering nodules of neoplastic tissue, there were discrete, round aggregates of mature lymphocytes often oriented near non-neoplastic vascular profiles (lymphoid follicle formation). Histologic features of metastatic disease in regional lymph nodes were similar to those of the primary neoplasm; metastases were expansile and caused widespread effacement of lymphoid architecture.

**Figure 4:**
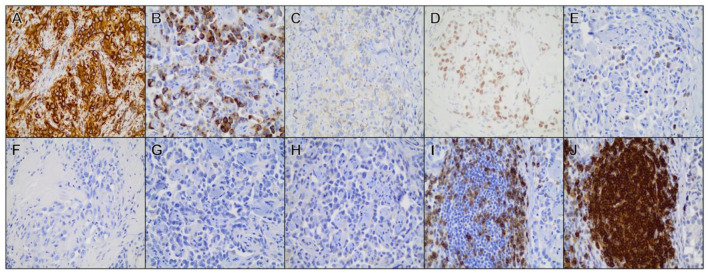
Immunohistochemical staining of representative tissue sections from the primary mass. Positive cytoplasmic/membranous labeling was observed for **(A)** vimentin, **(B)** factor VIII-related antigen, **(C)** CD31, **(D)** Prospero-related homeobox gene-1 (PROX-1). **(E)** Ki-67. **(F)** Neoplastic cells did not label for cytokeratin, **(G)** synaptophysin, **(H)** chromogranin-A. (**I)** Strong CD3 and **(J)** CD20 labeling was observed among lymphocytes forming discrete follicle-like structures at the periphery of the neoplasm. All photomicrographs are shown at × 40 magnification.

Additional IHC assessment was performed to further characterize the neoplasm, as well as discriminate between blood vessel and lymphatic endothelial origin (Figure 4). IHC was performed revealing positive cytoplasmic labeling of neoplastic cells for vWf (rabbit polyclonal, 1:1,000, Dako cat. A0082) and positive nuclear labeling for Ki67 (rabbit polyclonal, 1:100, Thermo cat. RM9106) in approximately 10% of cells. Inflammatory lymphoid follicles associated with the neoplastic tissues contained primarily CD20-positive B-lymphocytes (rabbit polyclonal, 1:300, Thermo cat. RB9013-P) with smaller numbers of intermixed CD3-positive T-lymphocytes (rabbit polyclonal, 1:200, Abcam cat. 5690). Neoplastic cells did not label for synaptophysin (mouse monoclonal, 1:500, Dako cat. M7315), chromogranin-A (rabbit polyclonal, 1:200, Abcam cat. 15160), or E-cadherin (rabbit monoclonal, 1:200, Cell Signaling cat. 3195). IHC performed on tissue sections sent to the CAHDC revealed positive nuclear labeling of neoplastic cells for PROX-1 (rabbit polyclonal, 1:500, Abcam cat. 38692).

It should be noted that all tissue samples were evaluated by multiple veterinary anatomic pathologists from two separate institutions (OSU and CSU) as well as a human pathologist associated with OSU. A diagnostic consensus of pleomorphic LAS with features of CHE was made based on the histopathologic evaluation and the IHC staining profile.

Based on the diagnosis of an angiosarcoma and the presence of loco-regional metastatic disease, adjuvant cytotoxic chemotherapy was recommended and pursued. Doxorubicin (30 mg/m^2^ IV; doxorubicin hydrochloride, Novaplus) was administered every 3 weeks for a total of five doses. Approximately 2 months after amputation, repeat thoracic radiographs showed no significant abnormalities. Approximately 4.25 months after amputation, repeat staging with thoracic radiographs and an abdominal ultrasound were performed. At this time no recurrence of the initial sarcoma or overt metastatic disease was observed. Approximately 7 months after surgery, a 4 × 0.5 cm firm, tubular, subcutaneous mass was observed over the previous limb amputation site. Fine needle aspirate confirmed the mass was a reoccurrence of the previously diagnosed malignancy of the animal, though repeated thoracic radiographs and abdominal ultrasound did not detect overt metastatic disease.

## Discussion

Here, we describe the clinical course along with the cytologic, histologic features in a case of pleomorphic LAS with features of CHE. LAS is an uncommon diagnosis in the dog, and while survival data are limited, improved patient outcomes have been reported following multimodal therapy (4). To date, only two cases of canine angiosarcoma with features of RH have been reported in the veterinary literature (15). Both of these cases had advanced disease present at the time of diagnosis. In the present case report, the primary neoplasm and local recurrence were localized to the skin, subcutis, and loco-regional lymph nodes, and distant metastatic disease was not observed. Furthermore, the tumor was reported to be growing on the dog's elbow for approximately 1.5 years prior to presentation, suggesting less aggressive biologic behavior. In humans, RH is a malignancy with a low-grade biologic activity that has been reported to metastasize on rare occasions (10). While most humans with RH do not have loco-regional metastatic disease present at the time of diagnosis, lymph nodes are the most common metastatic site (5, 12).

Significant discussion between the clinicians and pathologists involved in this case revolved around the pleomorphic appearance of the neoplasm, particularly given that the cytologic features in aspirates of the mass prior to biopsy prompted suspicion of carcinoma. Rarely, canine hemangiosarcoma may be subclassified as epithelioid or spindle-cell type based on significant deviation from histologic features considered typical of this neoplasm (6). While a diagnosis of EH would fit with the epithelial-like morphology of the neoplastic cells seen on both cytology and histology, the paucity of vascular profiles containing erythrocytes led us to also consider tumors of lymphatic endothelial origin as a differential diagnosis. Two candidate IHC antigens are reported to label lymphatic endothelium in veterinary species including canines: LYVE-1 and PROX-1 (7–9). Subsequent IHC labeling of tissue specimens from this case identified positive nuclear labeling for PROX-1 in neoplastic cells throughout the mass. IHC positivity for PROX-1, a transcription factor involved in lymphatic endothelial differentiation, supported modification of the initial diagnosis to LAS (16). PROX-1 labeling is not limited to lymphatic endothelial cells, as it may also be expressed in hepatocytes, lens fiber epithelium, intestinal endocrine cells, skeletal myocytes, and cardiomyocytes; however, it is not expressed by blood vascular endothelium (17).

Here, we report the first case of a canine LAS with features of CHE. PROX-1 IHC was used to confirm that the tumor arose from malignant lymphatics. In the two previously reported canine cases, only blood vessel endothelial markers (CD31 and vWf) were reported (15). While this tumor's positive labeling for PROX-1 supports classification of the angiosarcoma as a LAS, other subtypes of HE, such as Kaposiform HE and tufted angiosarcoma, have both been shown to express PROX-1 in people (18). Therefore, it is possible that the tumor described is a primary HE and not a primary LAS, although no substantial evidence on canine angiosarcoma or HE currently exists in the veterinary literature.

In conclusion, this report highlights the first reported case of canine LAS exhibiting CHE (features of EH and RH). In contrast to the previous two cases of RH in the dog, disease in the present case was more protracted. Angiosarcoma is a rare tumor type in domestic species; as a result, veterinary medicine currently lacks important understanding of the potential overlap in the clinical, histologic, and immunophenotypic features of the various vascular endothelial neoplasms falling under this categorical diagnosis. For this case, we use features of human vascular tumors to further characterize the neoplasm of interest; however, we are not suggesting that the human vascular neoplasm classification system would be an appropriate classification of dog vascular tumors. We merely are providing more information based on data that are available, which are predominantly human literature, in order to initiate more thorough evaluation and documentation of canine vascular tumors. Further reporting and characterization of veterinary vascular neoplasms is required to create an accurate classification system, which will eventually lead to appropriate implementation of treatment and improved understanding of typical biologic behavior.

## Data Availability Statement

The raw data supporting the conclusions of this article will be made available by the authors, without undue reservation.

## Author Contributions

MC wrote the clinical report and drafted the manuscript. JL and RC provided the anatomic pathology case write up, provided photomicrographs of the histopathology and IHC, and edited the manuscript. MW provided the clinical pathology case write up, provided photomicrographs of cytology for the case, and edited the manuscript. GZ and VW performed the surgery for the dog, provided information about the surgery that was performed, and edited the manuscript. WK and LS provided clinical insight and edited the manuscript. All authors read and approved the final manuscript.

## Conflict of Interest

The authors declare that the research was conducted in the absence of any commercial or financial relationships that could be construed as a potential conflict of interest.

## Publisher's Note

All claims expressed in this article are solely those of the authors and do not necessarily represent those of their affiliated organizations, or those of the publisher, the editors and the reviewers. Any product that may be evaluated in this article, or claim that may be made by its manufacturer, is not guaranteed or endorsed by the publisher.

## Abbreviations

CAHDC: Cornell Animal Health and Diagnostic Center
CHE: composite hemangioendothelioma
CT: computed tomography
EH: epithelioid hemangioendothelioma
HE: hemangioendothelioma
H&E: hematoxylin and eosin
IHC: immunohistochemistry
IV: intravenous
LAS: lymphangiosarcoma
LYVE-1: lymphatic vessel endothelium receptor-1
PROX-1: Prospero-related homeobox gene-1
RH: retiform hemangioendothelioma
vWf: von Willebrand factor.
